# Supplementary material for: Impact of living kidney donation on blood pressure and arterial stiffness: Systematic review and meta-analysis
Source: PLoS One. 2025 May 30;20(5):e0325390. doi: 10.1371/journal.pone.0325390 (PMC12124752; doi:10.1371/journal.pone.0325390)
Supplement: S2 Table — (PDF) [file pone.0325390.s002.pdf]

S2 Table. Assessment of the quality of studies included in the review was conducted according to the Newcastle-Ottawa Scale (NOS).

Study	Selection	Comparability	Outcome	NOS score
**Kasiske (2013)**	***	***	**	8/9
**Fesler (2015)**	***	**	***	7/9
**Moody (2015)**	***	**	***	8/9
**Kasiske (2015)**	***	*	***	7/9
**De Seigneux (2015)**	***	*	***	7/9
**Janki (2016)**	***	**	**	7/9
**Price-Earnest (2020)**	***	**	***	8/9
**Gokalp (2020)**	***	*	***	7/9
**Buss (2020)**	***	*	***	7/9
**Haugen (2020)**	***	**	**	7/9
**Price (2021)**	***	**	***	8/9
**Xagas (2023)**	***	**	**	7/9
