# Supplementary material for: Impact of living kidney donation on blood pressure and arterial stiffness: Systematic review and meta-analysis
Source: PLoS One. 2025 May 30;20(5):e0325390. doi: 10.1371/journal.pone.0325390 (PMC12124752; doi:10.1371/journal.pone.0325390)
Supplement: S3 Table — p = prospective, cs = case series. (PDF) [file pone.0325390.s003.pdf]

S3 Table. Basic characteristics of the trials included in the study. p= prospective, cs = case series

STUDY	Year	Country	Total (n)	Gender (male)	Age (mean)	Follow-up (yrs)	Trial Quality	GFR method measurement	BP method	Data extractors	Date of data extraction	Eligibilty of the study (NOS score)
Kasiske	2013	USA	203	65	43.4 (11.9)	0.5	p	CKD EPI	office BP	Manually	3/03/2024	8/9
Fesler	2015	France	45	6	51 (10)	1	p	99m TC-DTPA	office BP	Manually	3/03/2024	7/9
Moody	2015	UK	68	23	46.5 (12.1)	1	P	CKD EPI	mobil-ograph, & office BP	Manually	3/03/2024	8/9
Kasiske	2015	USA	182	58	43.4 (11.3)	3	p	CKD EPI	office BP	Manually	3/03/2024	7/9
De Seigneux	2015	Switzerland	21	9	54.1 (10.2)	1	p	CKD EPI	mobilograph	Manually	3/03/2024	7/9
Janki	2016	Nertherlands	176	82	52.8 (11.5)	5.1	p	CKD EPI	manually	Manually	3/03/2024	7/9
Price-Earnest	2020	UK	168	77	51 (12)	1	p	CKD EPI	mobilograph	Manually	3/03/2024	8/9
Gokalp	2020	Turkey	34	14	50.97 (13.04)	0.5	p	CKD EPI	NA	Manually	3/03/2024	7/9
Buss	2020	UK	51	23	49.5 (12)	1	p	51CrEDTA	mobilograph	Manually	3/03/2024	7/9
Haugen	2020	Norway	1029	453	44.8 (10.8)	11.3	cs	CKD EPI	manually	Manually	3/03/2024	7/9
Price	2021	UK	42	15	47.96 (12.49)	5	P	51CrEDTA	mobilograph, office BP	Manually	3/03/2024	8/9
Xagas	2023	Greece	40	11	56.8 (12.21)	1	p	CKD EPI	mobilograph	Manually	3/03/2024	7/9
